# Distribution and function of human long-lasting T cells in aging and non-small cell lung cancer

**DOI:** 10.3389/fimmu.2026.1788541

**Published:** 2026-07-15

**Authors:** Jia Xuan Tan, Hua Run Yu, Yong Gu, Hai Hong

**Affiliations:** 1 Key Laboratory of Tropical Disease Control of Sun Yat-Sen University, Ministry of Education, The Institute of Immunology of Zhong Shan Medical School, Sun Yat-Sen University, Guangzhou, China; 2 Department of Thoracic Surgery, The First Affiliated Hospital of Sun Yat-Sen University, Guangzhou, China

**Keywords:** antitumor immunity, immunometabolism, immunosenescence, lung cancer, memory T cell, T-cell dysfunction, tumor microenvironment

## Abstract

T lymphocytes, essential components of the adaptive immune system, orchestrate pathogen-specific responses and long-term immunological memory through heterogeneous subsets with distinct functional properties and spatial distributions. This comprehensive review delineates the origin, distribution, and functional specialization of long-lasting T cell subsets—naive T cells (Tn), stem cell-like memory T cells (Tscm), central memory T cells (Tcm), effector memory T cells (Tem), and tissue-resident memory T cells (Trm)—across physiological and pathological contexts, with a particular focus on aging and non-small cell lung cancer (NSCLC). We further dissect the complex intertwined mechanisms underlying immunosenescence and NSCLC pathogenesis. Elucidating how aging remodels T cell maintenance, tissue tropism and metabolic adaptation provides a conceptual framework for developing targeted therapeutic interventions against NSCLC.

## Introduction

The immune system operates as a complex and highly sophisticated defense network that protects the body against pathogenic invasion and maintains internal homeostasis through the coordinated actions of both the innate and adaptive immune systems. The innate immune system provides immediate and broad-spectrum defense against a wide variety of pathogens, whereas the adaptive immune system generates precise and long-lasting responses to specific threats through antigen specificity and immunological memory. The adaptive immune system consists of cellular and humoral immunity, among which T cell-mediated cellular immunity plays a crucial role in defending against recurrent infections and mediating anti-tumor immune responses.

**Figure 1:**
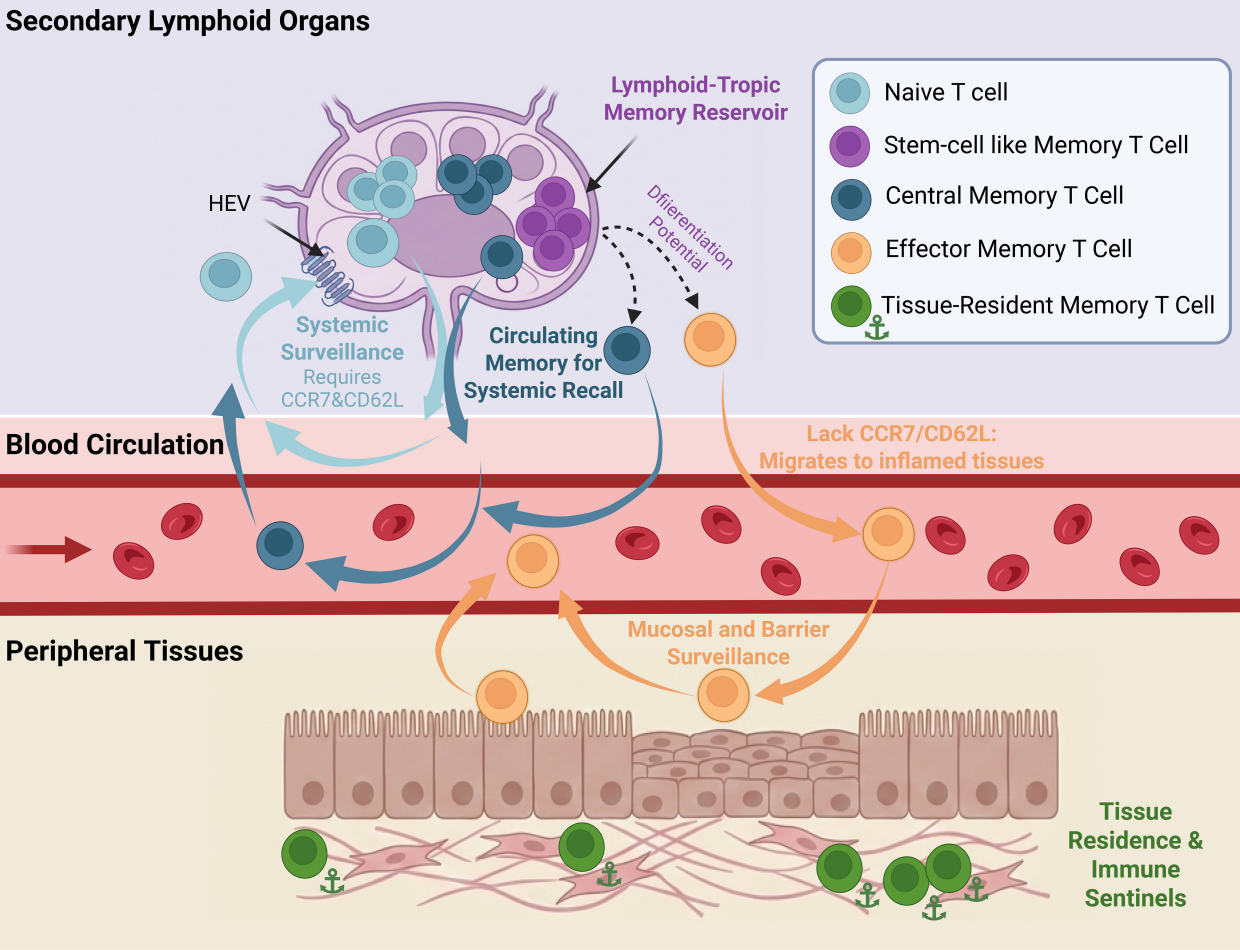
Schematic illustration of the migratory patterns and tissue distribution of human long-lasting T cell subsets. Naive T cells (Tn) and central memory T cells (Tcm) recirculate between the blood and secondary lymphoid organs via CCR7 and CD62L. Stem cell-like memory T cells (Tscm) exhibit lymphoid-homing properties and long-term self-renewal potential. In contrast, effector memory T cells (Tem) preferentially migrate to non-lymphoid peripheral tissues after downregulating CCR7 and CD62L. Tissue-resident memory T cells (Trm) are non-circulating cells retained within peripheral tissues and are characterized by expression of residency-associated markers. These distinct migratory programs underlie the compartmentalization and functional specialization of human long-lasting T cell subsets.

Naive T cells develop in the thymus and continuously recirculate between the bloodstream and secondary lymphoid tissues to detect cognate antigens (1). Upon activation by antigen-presenting cells (APCs), naive T cells undergo clonal expansion and differentiation, giving rise to effector T cells (Teff) that mediate immediate immune clearance. These effector cells eliminate infected or abnormal cells during the immune response. After antigen clearance, more than 95% of Teff cells undergo activation-induced cell death (AICD), while fewer than 5% survive and differentiate into long-lived memory T cells. Memory T cells are essential for long-term adaptive immunity, allowing rapid and robust responses to previously encountered antigens. Based on phenotype, function, and tissue-homing properties, memory T cells can be broadly classified into stem cell-like memory T cells (Tscm), central memory T cells (Tcm), effector memory T cells (Tem), and tissue-resident memory T cells (Trm). In the absence of antigenic stimulation, naive T cells and memory T cell subsets persist in a quiescent state while maintaining long-term persistence *in vivo*. Despite differences in antigen exposure, differentiation status, and functional specialization, these two T cell populations—both endowed with robust proliferative capacity—are unified by their hallmark attribute of longevity. Herein, we define naive and memory T cells collectively as long-lasting T (LLT) cells, a conceptual framework that expands beyond the traditional classification of memory T cells. Importantly, each LLT subset displays a unique migratory signature that is tightly intertwined with its immunological function (**Figure 1**). This spatial partitioning facilitates efficient immune surveillance and rapid responses across diverse tissue compartments.

The distribution and function of long-lasting T cell subsets are dynamic, influenced by intrinsic host factors and external environmental cues. Age is a key intrinsic regulator that shapes T-cell homeostasis throughout life. In addition, extrinsic pathological conditions—including malignancies, can also reshape the composition and function of T cell subsets, contributing to tumor progression, metastasis, and poor clinical outcomes. Elucidating the spatiotemporal distribution and functional heterogeneity of long-lasting T cells is crucial for advancing our understanding of human immune biology and improving clinical interventions for immune-related disorders.

## The distribution and function of Tn cells

Naive T cells (Tn) arise from bone marrow-derived thymic seeding progenitors (TSPs), which migrate to the thymus and undergo a tightly regulated, multistep developmental program. T cells development starts with the differentiation of TSPs into double-negative (DN; CD4^-^CD8^-^) thymocytes. Following successful pre-TCR rearrangement and expression, consisting of the TCR β-chain and invariant pre-Tα chain, thymocytes upregulate CD4 and CD8 and become double-positive (DP; CD4^+^CD8^+^) thymocytes. DP thymocytes then undergo positive selection, differentiate into single-positive (SP; CD4^+^ or CD8^+^) thymocytes and acquire major histocompatibility complex (MHC) restriction. Subsequently, in the thymic medulla, SP thymocytes undergo negative selection to establish self-tolerance. Ultimately, only approximately 1–5% of thymocytes survive these selection processes and exit the thymus as mature antigen-naive T cells. After thymic egress, Tn cells migrate to thymus-dependent regions of secondary lymphoid organs (SLOs), including the lymph node paracortex, the periarteriolar lymphoid sheaths (PALS) of the spleen, and the interfollicular regions of gut-associated lymphoid tissue (GALT). Within these niches, Tn cells localize in proximity to antigen-presenting dendritic cells (DCs), thereby facilitating efficient antigen recognition and priming for subsequent responses to foreign antigens (2, 3). Tn cells continuously recirculate between blood and secondary lymphoid organs every 12–24 hours. This process is directed by the homing receptors CD62L and CCR7, which mediate trafficking through interactions with ligands expressed on high endothelial venules (HEVs). Such dynamic recirculation maximizes encounters with rare antigen-specific clones and maintains T-cells diversity and immune homeostasis (1, 4, 5).

## The impact of aging on Tn cells

**Figure 2:**
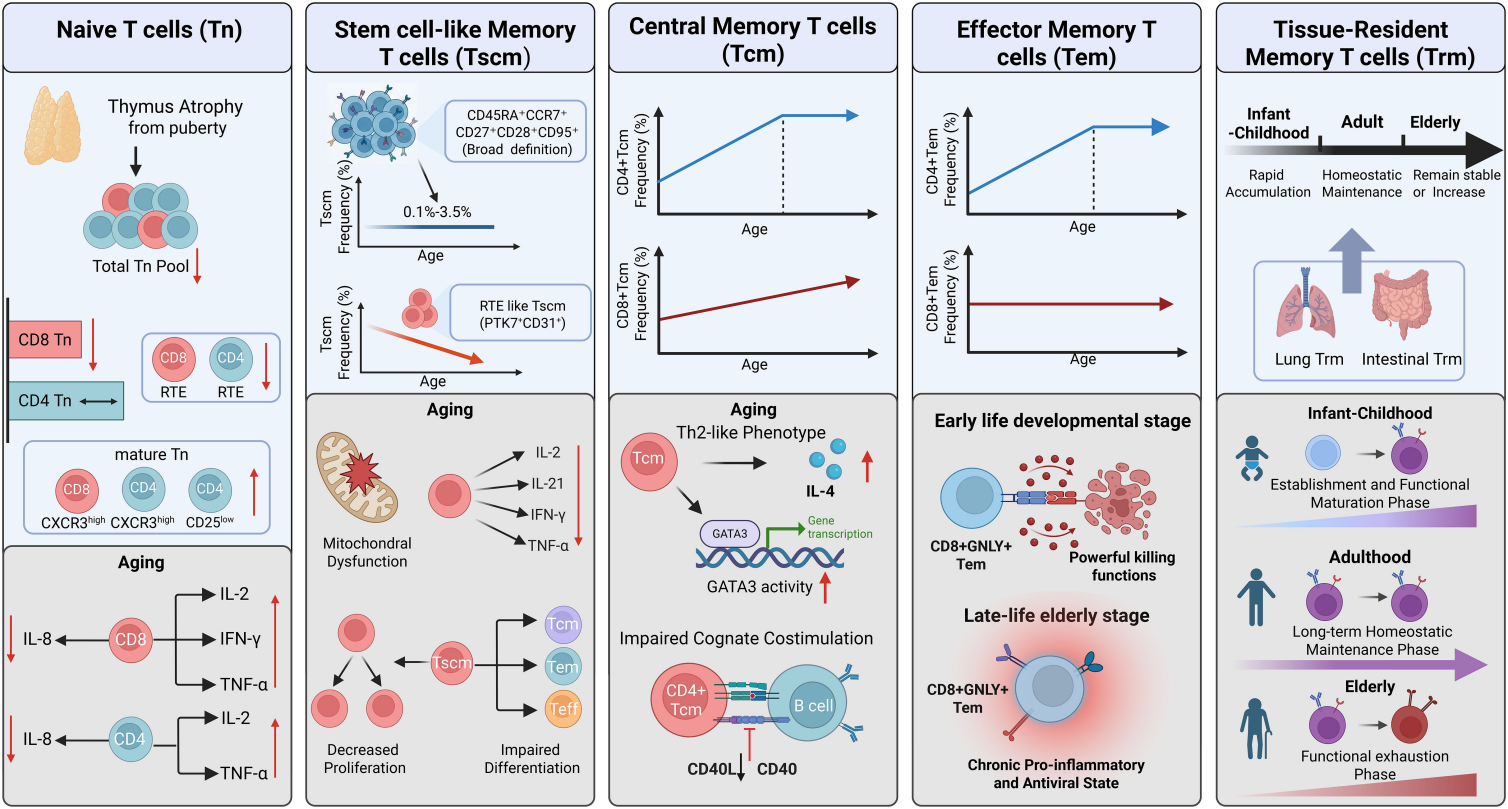
Age-related alterations in the distribution and function of human long-lasting T-cell subsets. Aging differentially affects the abundance and function of long-lasting T-cell subsets. Thymic involution reduces the naive T-cell (Tn) pool, especially in the CD8^+^ compartment. Stem cell-like memory T cells (Tscm) undergo remodeling, with depletion of precursor-like subsets and reduced proliferative fitness. Central memory T cells (Tcm) accumulate and acquire a Th2-biased profile, whereas effector memory T cells (Tem) shift toward chronic inflammatory and antiviral states with age. Tissue-resident memory T cells (Trm) display early accumulation, stable maintenance in adulthood, and functional exhaustion later in life.

Thymic involution represents a central feature of immunological aging (6), with thymic function declining substantially from puberty onward (7). This progressive deterioration reduces thymic output and compromises maintenance of the naive T-cells (Tn) compartment (8, 9). Consequently, the proportion of naive CD8^+^ T cells declines markedly with age, whereas that of naive CD4^+^ T cells remain comparatively stable (10–13). Consistent with reduced thymopoiesis, CD38^high^ recent thymic emigrants (RTEs) are markedly decreased within the Tn compartment (14). Furthermore, the composition of the residual naive pool shifts, characterized by an age-related increase in CXCR3^high^ cells among both naive CD8^+^ and naive CD4^+^ T cells, while CD25^low^ cells specifically accumulate within the naive CD4^+^ T cell population (14). These compositional changes are associated with a shift toward a pro-inflammatory functional profile. In both CD8^+^ and CD4^+^ naive T cells, IL-8 secretion is reduced, whereas IL-2 and TNF-α production are increased; in contrast, enhanced IFN-γ production is restricted to the naive CD8^+^ subset (14). Taken together, thymic involution not only reduces naive T-cell output but also remodels the composition of the remaining Tn pool and promotes a functional shift toward a pro-inflammatory state (**Figure 2**). These changes underscore the complexity of immunosenescence and highlight the importance of subset-specific analyses of naive T cells.

## The composition and function of Tn cells in NSCLC

Malignancies significantly impact naive T cell dynamics. Single-cell transcriptomic studies have shown reduced frequencies of peripheral naive T cell in patients with breast cancer, head and neck squamous cell carcinoma, pancreatic ductal adenocarcinoma, renal cell carcinoma, and urothelial carcinoma (15). Flow cytometric analyses further support a decline in circulating naive CD4^+^ T cells across multiple cancers (16). Although these observations suggest that contraction of the peripheral Tn pool may represent a common feature of cancer-associated immune dysregulation, most available studies are cross-sectional, and the relative contributions of tumor burden, chronic antigen exposure, systemic inflammation, and host age remain difficult to disentangle.

**Figure 3:**
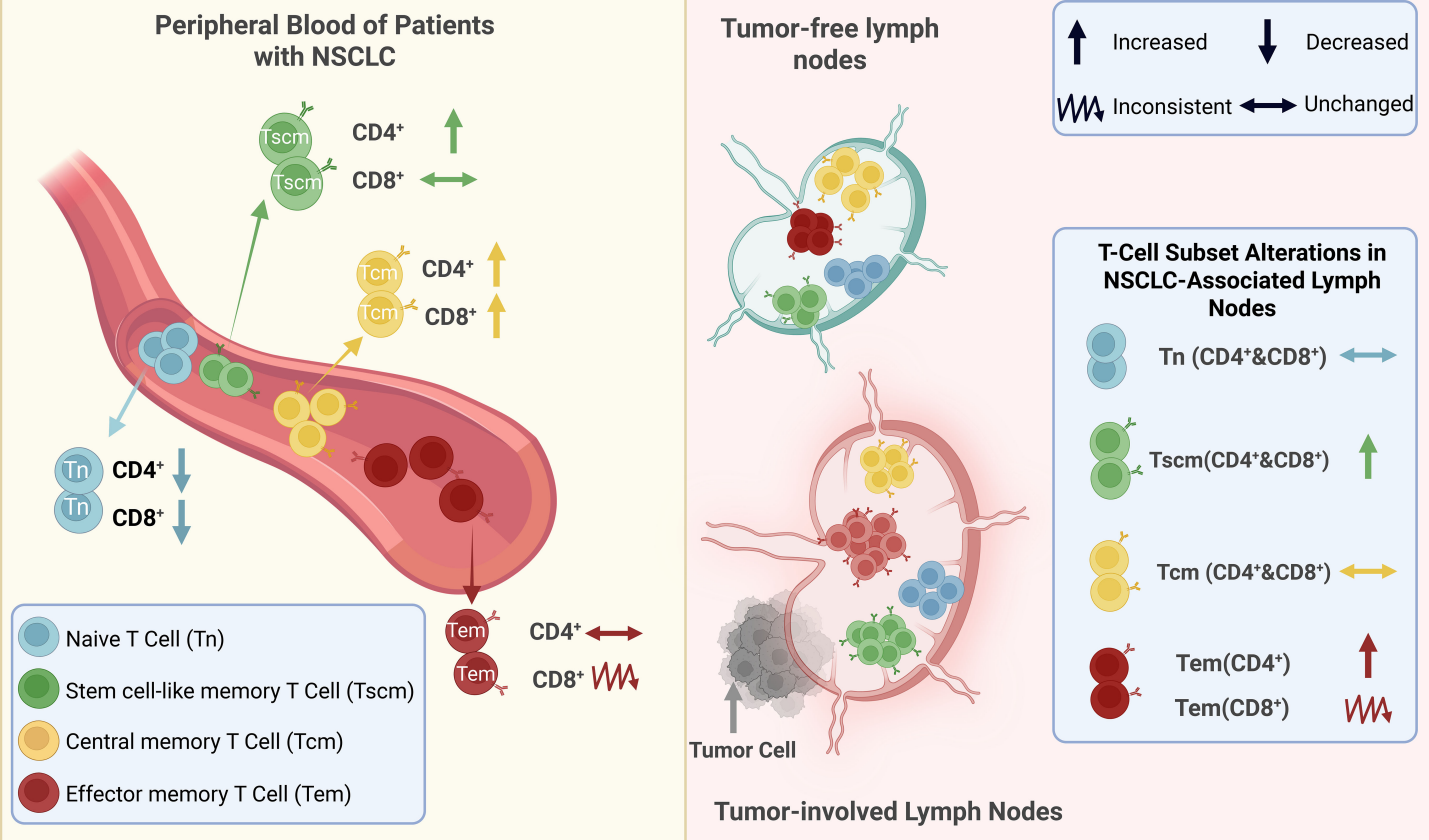
Distribution of long-lasting T-cell subsets in peripheral blood and tumor-involved lymph nodes of patients with NSCLC. Compared with healthy individuals, patients with NSCLC show reduced frequencies of both CD4^+^ and CD8^+^ naive T (Tn) cells in peripheral blood, accompanied by selective expansion or relative enrichment of memory subsets, particularly CD4^+^ Tscm and Tcm cells. In tumor-involved lymph nodes, Tn-cell frequencies remain largely comparable to those in tumor-free nodes, whereas both CD4^+^ and CD8^+^ Tscm cells are increased. Tcm frequencies show little overall change, while CD4^+^ Tem cells become relatively enriched.

In NSCLC, both CD4^+^ and CD8^+^ Tn compartments appear to be quantitatively affected (**Figure 3**). Peripheral naive CD4^+^ T cells decline progressively from early- to late-stage disease (17), and reduced frequencies of naive CD8^+^ T cells have also been reported in the peripheral blood of patients with lung cancer (18). Notably, a higher CD4^+^ naive-to-memory ratio has been associated with longer progression-free survival and identified as an independent favorable prognostic factor (19), supporting the potential clinical relevance of preserved naive T-cell homeostasis. However, whether the decline in circulating Tn cells directly contributes to tumor progression or simply reflects broader immune remodeling remains unresolved. Functional alterations in NSCLC Tn cells add another layer of complexity. Peripheral CD8^+^ Tn cells from patients with NSCLC show reduced IFN-γ and TNF-α production, whereas CD4^+^ Tn cells display impaired TNF-α secretion without significant changes in IFN-γ (20). These findings point to subset-specific dysfunction, but they also raise an interpretive challenge: because naive T cells are generally characterized by limited immediate effector cytokine production, the biological meaning of altered cytokine readouts in this compartment requires careful consideration. Importantly, Tn-cell abnormalities in NSCLC do not appear to be uniform across anatomical sites. In tumor-involved lymph nodes, both the frequency and cytokine production of CD4^+^ and CD8^+^ Tn cells were reported to be comparable to those in tumor-free lymph nodes (20, 21). This discrepancy between peripheral blood and lymphoid tissues suggests that tumor-associated effects on Tn cells may be compartment-specific rather than systemic in a uniform manner. At the same time, the current evidence remains limited, and it is still unclear whether these apparently preserved lymph node Tn cells are functionally equivalent in terms of proliferative capacity, differentiation potential, and antigen responsiveness.

Within the tumor microenvironment, tumor-infiltrating lymphocytes (TILs) are generally regarded as indicators of ongoing antitumor immunity, and their abundance and phenotypic characteristics often correlate with improved clinical outcomes (22–24). However, the presence of naive-phenotype T cells in tumors remains conceptually and experimentally difficult to interpret, given that persistent antigen exposure would be expected to limit the retention of truly antigen-inexperienced lymphocytes. Consistent with this, naive T cells have been reported in tumor infiltrates only at low frequencies relative to peripheral tissues (25–27). In NSCLC, both CD4^+^ and CD8^+^ Tn subsets are markedly underrepresented in tumors compared with peripheral blood or adjacent non-malignant lung tissue (25). Although CD45RA^+^CCR7^+^ cells have been identified among NSCLC TILs (28), whether these cells represent bona fide naive T cells remains unresolved. Their high IFN-γ production and elevated CD95 expression are difficult to reconcile with a resting naive state and instead suggest prior antigen experience (28, 29). These findings highlight an important limitation in the field: conventional phenotypic markers may be insufficient to distinguish true Tn cells from activated, stem-like, or reverted memory populations within tumors. More stringent phenotypic and functional criteria will therefore be required to clarify whether bona fide naive T cells truly persist in the NSCLC microenvironment.

Overall, current evidence suggests that aging and NSCLC reshape the Tn-cell compartment in terms of abundance, function, and tissue distribution, but the precise identity and role of these cells in antitumor immunity remain to be fully defined.

## The distribution and function of Tscm cells

Tscm cells occupy a unique position within the memory T-cell hierarchy, characterized by a naive-like phenotype, long-term self-renewal capacity, and multipotent differentiation into downstream memory and effector T-cell subsets (30). Initially identified in mice as a rare CD44^low^CD62L^high^CD8^+^ T cell subset with stem cell-like properties—longevity and the ability to reconstitute memory and effector T cells (31)—similar populations were later confirmed in humans (30). Human Tscm cells display a unique combination of naive-like markers (CD45RA, CCR7, CD62L, CD27, CD28, IL-7Rα) and elevated expression of memory- and activation-associated molecules (CD95, IL-2Rβ, CXCR3, CD58, LFA-1) (30, 32, 33). In addition to these surface markers, Tscm cells exhibit a transcriptional profile associated with stem-like properties and long-term persistence, including the expression of TCF1, LEF1, FOXP1, and BCL2 (30). This combination of stemness and functional competence enables Tscm cells to support immune homeostasis, act as a durable reservoir for immune reconstitution, and contribute to both protective immunity and immunopathology.

The generation and maintenance of Tscm cells are regulated by antigenic priming, microenvironmental cues, and intracellular signaling. Current evidence suggests that these cells arise from naive T cells following antigen exposure, with their differentiation influenced by both tissue localization and signaling context (34). For example, during viral infection, CXCR3 directs CD8^+^ effector T cells to peripheral regions of the lymph node, whereas CXCR3 deficiency results in their retention in more central nodal areas, thereby favoring the generation of stem-like memory precursors (35). *In vitro* studies have shown that stimulation in the presence of Wnt3A or glycogen synthase kinase-3β (GSK-3β) inhibitors effectively promotes the differentiation of naive CD8^+^ T cells into Tscm cells (36). Notch signaling can drive CD4^+^ T cells toward a Tscm-like state with a naive-like phenotype (CD44^low^CD62L^high^) (37). Additionally, treatment with TWS119 converts CD4^+^ T cells into Tscm cells (38). Cytokine conditioning with IL-7 and IL-15 during *in vitro* culture promotes the generation of T memory stem cells (Tscm) (39).

Tscm cells display a tissue distribution similar to that of naive T cells, with a strong preference for secondary lymphoid organs. In both nonhuman primates and humans, they primarily localize to lymph nodes (LNs) and the spleen, with limited representation in peripheral tissues (30, 40). Studies in nonhuman primates (NHPs) have demonstrated that CD8^+^ Tscm cells are highly enriched in lymph nodes (LNs) but are virtually absent from mucosal tissues and detectable only at low frequencies in the spleen and bone marrow (40). Despite their remarkable longevity—persisting for 12–25 years in humans without clear evidence of senescence (41, 42)—Tscm cells remain exceptionally rare in peripheral circulation. In blood, they account for only approximately 2–3% of both CD4^+^ and CD8^+^ T cells, a significantly lower proportion than conventional memory subsets such as Tcm and Tem (30). This scarcity is further highlighted by their low frequency in umbilical cord blood (<1%) (30). Together, these findings indicate that Tscm cells are a rare T-cell subset with a distribution pattern biased toward secondary lymphoid organs rather than peripheral blood or non-lymphoid tissues.

## The impact of aging on Tscm cells

The impact of aging on Tscm cells is complex and depends on methodological and phenotypic definitions. When a relatively broad phenotype is used, with Tscm identified as CD45RA^+^CCR7^+^CD27^+^CD28^+^CD95^+^, no significant age-associated differences in Tscm frequency are observed in either the CD4^+^ or CD8^+^ compartment, suggesting that the overall circulating Tscm pool may be numerically preserved during aging (32, 43, 44). In contrast, higher-resolution phenotyping using markers such as CCR7, CD45RO, CD27, CD62L, CD95, CD122, CD31, and PTK7 has shown that CD4^+^ Tscm cells decline with age, with a more pronounced reduction in the (PTK7^+^CD31^+^) recent thymic emigrant-like (RTE-like) subset (45). This reduction is associated with thymic involution and decreased precursor availability (45), suggesting that aging may preferentially erode the more stem-like and less differentiated fractions of the Tscm compartment rather than uniformly reducing the entire population. These discrepancies across studies likely reflect differences in cohort composition, flow cytometric gating strategies, and the degree of age stratification. In addition, the lack of epigenetic analyses remains an important gap, as aging-related epigenetic alterations may reshape Tscm function without necessarily changing their overall frequency.

More detailed analyses further indicate that aging is accompanied by qualitative deterioration of Tscm, particularly within the CD4 compartment. In this context, CD4^+^ Tscm from older individuals exhibit reduced proliferative and differentiation capacity, diminished secretion of IL-2, IL-21, IFN-γ, and TNF, and a decline in TCR repertoire diversity (45). In parallel, these cells show signs of mitochondrial and metabolic disturbance, including reduced average mitochondrial volume, abnormal cell polarity, and altered metabolic programming (45). Such changes may reflect a gradual loss of mitochondrial homeostasis and bioenergetic fitness, thereby limiting the ability of Tscm to sustain self-renewal and long-term functional maintenance. These age-related defects are further accompanied by attenuation of canonical Wnt/β-catenin signaling, reflected by reduced TCF-1 expression, increased circulating DKK-1, decreased SFRP1, and elevated autoantibodies against proteins involved in the Wnt pathway (45).

More broadly, aging remodels T-cell transcriptional networks, shifting them from programs that support quiescence, self-renewal, and plasticity toward those favoring effector differentiation, terminal maturation, and exhaustion-like states. Accordingly, T cells from younger individuals express higher levels of stemness-associated transcription factors, including TCF7/TCF1, LEF1, FOXP1, and IKZF1, whereas aging is accompanied by reduced activity of these factors and increased expression of EOMES, TBX21, RUNX3, and NFATC2 (46). Within this broader context, Tscm cells may serve as a particularly informative population for tracking the age-related loss of T-cell stemness at phenotypic, functional, and transcriptional levels.

Taken together, current evidence indicates that aging reshapes the Tscm compartment at multiple levels, particularly by altering subset composition and impairing functional integrity, while changes in the overall frequency of broadly defined Tscm populations may be less apparent (**Figure 2**). This distinction is critical for interpreting the literature and supports the view that Tscm dysfunction may contribute to age-related immune decline and could represent a target for immune rejuvenation strategies.

## The composition and function of Tscm cells in NSCLC

In NSCLC, Tscm cells undergo a profound redistribution across the peripheral blood, tumor-draining lymph nodes, and tumor microenvironment (**Figure 3**). This altered spatial distribution is coupled with compartment-specific functional adaptation. Relative to healthy donors, NSCLC patients display a selective expansion of circulating CD4^+^ Tscm cells (2.59% vs 1.74%), whereas the frequency of CD8^+^ Tscm cells remains unaltered (47) in blood. This observation stands in contrast to a previous study reporting coordinated changes in both T-cell subsets (48), underscoring the importance of accounting for cohort heterogeneity—including tumor stage and prior therapeutic exposure—as potential contributors to such divergent findings. Tumor-infiltrated lymph nodes exhibit marked Tscm expansion, with CD8^+^ and CD4^+^ Tscm cells at 18.99% and 29.91%, respectively, compared to much lower levels in healthy lymph nodes (CD8^+^ Tscm–1.25%; CD4^+^ Tscm–4.88%) (47). This observation aligns with the established function of tumor-involved lymph nodes as reservoirs for tumor-reactive T cells. Nonetheless, it remains unresolved whether this expansion arises from augmented *de novo* generation of Tscm cells, diminished differentiation toward effector T cell subsets, or impaired egress of these cells into the tumor microenvironment. Disentangling these mechanisms will be critical for defining the functional relevance of Tscm accumulation within this anatomical compartment. Within the TME, NSCLC tumor-infiltrating lymphocytes (TILs) also harbor substantial Tscm populations (CD4^+^–12.08%; CD8^+^–9.87%) (28). While Tscm cell frequencies are lower within TILs than in lymph nodes, they remain substantially enriched relative to peripheral blood. Given these feasibility considerations for adoptive immunotherapy, TIL-based cellular therapy therefore exhibits favorable translational potential in clinical settings.

In parallel with these compositional changes, Tscm cells in NSCLC also exhibit compartment-dependent functional remodeling. CD4^+^ Tscm cells show increased IFN-γ production, especially in peripheral blood (HD: 30.7% vs. NSCLC: 55.49%) and lymph nodes (health: 3.94% vs. tumor-involved: 11.55%) (47). An even higher frequency was observed in TILs, where IFN-γ^+^CD4^+^ Tscm cells reached 77.61% (47). Whether this heightened IFN-γ production is sustained over time or diminishes with disease progression, limiting our understanding of Tscm-mediated anti-tumor immunity. Conversely, CD8^+^ Tscm cells show consistent IFN-γ^+^ frequencies between TILs and peripheral blood, indicating subset-specific regulation within the tumor microenvironment (28, 47). This spatial heterogeneity underscores the ability of Tscm cells to adapt to distinct immunological environments during cancer progression, yet integrative analyses linking these functional adaptations to clinical outcomes (e.g., patient survival, treatment response) remain scarce—emphasizing the need for more cohesive literature synthesis to translate these observations into actionable insights for NSCLC immunotherapy.

Taken together, these findings indicate that NSCLC reshapes both the distribution and effector properties of Tscm cells in a compartment-dependent manner. Their preferential enrichment and functional activation in tumor-associated compartments support the view that Tscm cells are not merely a reservoir of stem-like memory, but an active component of the antitumor immune response in NSCLC.

## Immunotherapy based on Tscm cells

Tscm cells provide long-term antitumor protection by persisting, proliferating, and generating effector CD8^+^ T cells (32, 49). Immunotherapy based on tumor-responsive T cells has shown considerable therapeutic potential, but its clinical benefit remains limited by unsatisfactory response rates (50, 51). Better therapeutic outcomes are associated with infused T cells exhibiting long telomeres or high expression of CD62L, CD28, or CD27, highlighting the importance of expanding less differentiated populations such as Tscm cells (30, 36, 52–54). IL-7 and IL-15 promote Tscm cells development and expansion, while IL-21 enhances their proliferation and antitumor function (39, 55). In combination with IL-21, the IL-2 partial agonist H9T sustains stem-like CD8^+^ T cells with enhanced anticancer activity (56).

Adoptive cellular immunotherapy has provided a major framework for the therapeutic application of Tscm cells. Chimeric antigen receptor (CAR) T-cell therapy has demonstrated substantial clinical efficacy and has become a key treatment modality for B-cell malignancies (57). Studies in mice have shown that generating CAR T cells from immature T cells or Tscm cells enhances antitumor responses while reducing cytokine release syndrome (CRS) (58).Moreover, supplementing IL-15 during CAR T-cell expansion preserves the Tscm cell phenotype and inhibits mTORC1 activity, thereby improving metabolic fitness and antitumor efficacy (59). Expanding Tscm cells within TILs is also important for boosting antitumor activity. It has been reported that blocking phosphatidylinositol 3-kinase delta (PI3Kδ) in both CAR T-cell therapy and TIL-based adoptive cell therapy (ACT) increased Tscm cell generation, thereby enhancing cellular metabolism and antitumor immunity (60). Since CD8**^+^** T-cell differentiation involves metabolic reprogramming, the regulation of metabolic pathways is crucial for modulating antitumor immunity (61). Several *in vitro* and *in vivo* studies have shown that metabolic interventions can promote Tscm generation and enhance CAR T-cells efficacy. Rapamycin-mediated mTOR inhibition has been reported to induce Tn-to-Tscm differentiation, accompanied by a metabolic shift toward fatty acid oxidation (FAO) (38). Another study has demonstrated that rapamycin treatment enhanced mitochondrial function in CAR-T cells, leading to improved infiltration into solid tumors and better tumor control (62). Short-term inhibition of the mitochondrial pyruvate carrier (MPC) promoted memory-like differentiation, and improved the antitumor activity as well as functional persistence of CAR-T cells during manufacturing (63). Exogenous metabolic supplementation with β-hydroxybutyrate (BHB) enhances CAR-T cell proliferation, cytokine production, and tumor control (64). Collectively, these findings support metabolic modulation as a promising strategy to optimize both Tscm generation and CAR-T cell immunotherapy.

## The distribution and function of Tcm and Tem cells

Immunological memory is a hallmark of adaptive immunity, enabling rapid and robust responses upon re-exposure to previously encountered antigens. The origin of memory T cells remains debated: they may arise either from a small subset of effector T cells that survive the contraction phase or from precursors exposed to relatively low levels of antigen that subsequently differentiate into memory cells (65, 66). In addition, stem cell-like memory T cells, characterized by self-renewal capacity, are thought to give rise to both central memory and effector memory T-cell subsets (30). Central memory T cells (Tcm), defined by the expression of CCR7 and CD62L, primarily recirculate between the blood and secondary lymphoid organs, where they function as a reservoir for systemic recall response. In contrast, effector memory T cells (Tem) lack these lymphoid-homing receptors and preferentially migrate to peripheral tissues (67).

Comprehensive tissue-mapping studies have demonstrated that Tcm (CD45RO^+^CCR7^+^CD62L^+^) and Tem (CD45RO^+^CCR7^-^CD62L^+^/^-^) cells show distinct anatomical distributions consistent with their respective immunological roles (68, 69). Utilizing broadly comparable phenotypic definitions across multicolor flow cytometry and immunohistochemical analyses, these studies consistently demonstrated that central memory T (Tcm) cells are predominantly enriched within lymphoid and circulatory compartments, including peripheral blood, spleen, and lymph nodes (68, 69). Their abundance also differs between CD4^+^ and CD8^+^ T-cell lineages: among CD4^+^ T cells, Tcm cells represent a relatively stable and substantial population (approximately 20–30%) in blood, lymph nodes, and lung, whereas CD8^+^ Tcm cells remain relatively infrequent, generally accounting for less than 10% across tissues (68, 69). This lineage-specific disparity may reflect differences in developmental programs, homeostatic demands, or functional specialization between the CD4^+^ and CD8^+^ memory T-cell compartments; however, this interpretation necessitates further comprehensive analysis. By contrast, Tem cells are the predominant memory subset in mucosal tissues such as the lung, jejunum, ileum, and colon, and are also abundant in the spleen (68, 69). Although present in blood and lymph nodes, Tem cells are generally detected at lower frequencies in these lymphoid compartments than in mucosal tissues (68, 69). This tissue-selective enrichment is consistent with the role of Tem cells in mediating rapid effector responses at barrier sites, where they contribute to early protection against reinfection. Further analysis of lymphoid compartments has revealed two additional patterns: first, Tem cells may outnumber Tcm cells in peripheral blood and lymph nodes, particularly within the CD8^+^ compartment; second, both CD4^+^ Tcm and CD4^+^ Tem cells are generally more abundant than their CD8^+^ counterparts in these tissues (20). Although some variability among studies may be attributed to technical factors, such as tissue processing protocols and the specific antibody clones employed in flow cytometry, as well as sampling-related variables including donor age, health status, and timing of tissue collection, the general pattern of tissue-specific compartmentalization of Tcm and Tem cells remains largely consistent.

## The impact of aging on Tcm and Tem cells

Human T-cell compartments undergo dynamic age-related remodeling driven by cumulative antigen exposure and thymic involution (69). Evidence derived from flow cytometric, transcriptional, and functional analyses conducted across multiple independent cohorts consistently substantiates the age-associated alterations in T-cell composition and function, notwithstanding some variability in phenotypic definitions among the studies. Both CD4^+^ and CD8^+^ T cells progressively shift from a naïve-dominant to a memory-dominant phenotype with age, with this transition occurring at approximately 37.4 years for CD4^+^ T cells and 29.5 years for CD8^+^ T cells (70). The earlier transition observed in CD8^+^ T cells aligns with evidence indicating that the CD8^+^compartment is more strongly influenced by persistent antigenic stimulation and is more susceptible to progressive differentiation and exhaustion in contexts of chronic viral infection and aging, although the timing of these changes may differ among cohorts (71–73). Among CD4^+^ T cells, both Tcm and Tem subsets increase with age and tend to stabilize after 65 years. By contrast, CD8^+^ Tcm cells gradually accumulate over time and reach significantly higher levels in older adults than in younger individuals, whereas CD8^+^ Tem cells remain relatively stable (74). Together, these findings indicate distinct age-associated trajectories in memory T-cell subset composition across CD4^+^ and CD8^+^ lineages.

In addition to these quantitative changes, aging is accompanied by substantial functional remodeling of memory T cells. Tcm cells undergo stable transcriptional reprogramming and progressively acquire a Th2-like functional profile, characterized by enhanced GATA3 activity and increased IL-4 secretion (75). This shift is associated with reduced surface expression of CD40 ligand (CD40L) on CD4^+^ Tcm cells, thereby impairing their ability to provide co-stimulatory signals to cognate memory B cells and ultimately leading to attenuated antibody recall responses and skewed antibody class switching following antigen re-exposure, such as after influenza vaccination (75). Several lines of evidence from vaccine immunology suggest that age-related dysfunction within the CD4^+^ memory T-cell compartment—most notably compromised maintenance and function of Tcm cells—may underpin the diminished efficacy of influenza vaccines in older adults (76, 77). By comparison, Tem cells exhibit a biphasic pattern of functional adaptation with age. In childhood, clonally expanded CD8^+^GNLY^+^ Tem cells express high levels of cytotoxic molecules and display potent killing activity, whereas in older adults, these clonally expanded CD8^+^GNLY^+^ Tem cells adopt a state marked by increased expression of interferon-related genes, consistent with a chronic inflammatory and antiviral phenotype (78). More broadly, aging is associated with reduced TCR diversity, a transcriptomic shift toward a pro-inflammatory state, and extensive rewiring of intercellular communication networks, particularly those involving immune checkpoint molecules (78). Collectively, these quantitative and functional alterations define the progressive remodeling of memory T cells during immunosenescence and contribute to the age-related decline in adaptive immune function (**Figure 2**).

## The composition and function of Tcm and Tem cells in NSCLC

Lung cancer is associated with remodeling of the peripheral memory T-cell compartment, although the reported changes are not fully consistent across studies (**Figure 3**). This remodeling is most clearly reflected by a selective expansion of CD4^+^ memory T cells, indicative of antigen-driven activation, whereas a consistent increase in total CD8^+^ memory T cells has not been observed (16, 17, 19, 79). At the subset level, the available evidence points to dynamic but heterogeneous changes rather than a uniform pattern. In NSCLC, CD4^+^ Tcm cell frequencies show an increasing trend (80), and elevated frequencies of CD8^+^ Tcm and Tem cells have also been reported (18); similarly, increased CD8^+^ Tcm and Tem subsets have been described in head and neck squamous cell carcinoma (81, 82). Notably, this remodeling is not unidirectional, lower proportions of CD8^+^ Tem cells among total CD8^+^ T cells have also been reported in NSCLC (20), with a similar reduction observed in malignant melanoma (83). In contrast, the distribution of CD4^+^ Tem cells remains unchanged in NSCLC compared to healthy control (20). These findings suggest that peripheral memory T-cell alterations in cancer reflect a dynamic, subset-specific remodeling process rather than a fixed immunophenotypic shift, with the direction of change varying according to study design, patient cohorts, and methodological approaches to T-cell subset phenotyping. Dynamic alterations in memory T cell subsets are a consistent feature across diverse tumor types, with distinct patterns of subset distribution associated with tumor stage and therapeutic intervention. In colorectal cancer, advanced-stage (III–IV) disease is characterized by a reduced proportion of CD8^+^ Tcm cells relative to early-stage (I–II) disease (84), whereas in head and neck squamous cell carcinoma, advanced-stage (III–IV) patients exhibit lower frequencies of effector memory T (Tem) cells compared to their early-stage counterparts—findings that imply progressive differentiation of memory T cells as tumors advance (82). Notably, in gastric cancer, the observed increase in circulating Tem cells and reduction in Tcm proportions are reversed following tumor resection (85), providing direct evidence that the tumor microenvironment actively drives the differentiation of Tcm toward the Tem phenotype. Overall, these observations underscore the critical role of memory T cell subset dynamics in tumor progression and their potential as both prognostic biomarkers and therapeutic targets. Future studies should focus on unraveling the molecular mechanisms underlying tumor-induced memory T cell differentiation, as well as exploring strategies to modulate these subsets to enhance anti-tumor immunity and improve clinical outcomes for cancer patients.

Spatial context further contributes to this complexity. In tumor-involved versus uninvolved lymph nodes, memory T-cell distributions also show both shared and cancer-specific patterns. In NSCLC, the frequencies of CD4^+^ and CD8^+^ Tcm cells are similar between tumor-draining and normal lymph nodes (20). However, CD4^+^ Tem cells predominate in tumor-draining nodes, exceeding levels in tumor-free nodes and peripheral blood, whereas CD8^+^ Tem cells frequencies remain unchanged (20). In breast cancer, tumor-involved lymph nodes show a decrease in CD4^+^ Tem cells and an increase in Tcm cells (86, 87). In bladder cancer, CD8^+^ Tcm cells constitute the predominant CD8^+^ memory T cell subset in tumor-draining lymph nodes, and their frequency increases significantly with disease progression (88). These findings reinforce the notion that tumor-draining lymph nodes act not merely as passive cellular reservoirs, but as pivotal sites driving tumor-specific modulation of Tcm and Tem cell populations.

A further layer of heterogeneity is evident within the tumor itself. Within the tumor microenvironment, CD4^+^ and CD8^+^ memory T-cell subsets display distinct distributions in TILs from NSCLC patients (28). Among CD4^+^ TILs, Tem cells are the most abundant subset (56.08%), followed by Tcm cells (21.85%). In contrast, CD8^+^ TILs are predominantly composed of Tem (37.65%) and Teff (33.99%) cells, with Tcm cells being the least abundant (11.75%) (28). Notably, the proportions of both Tem and Tcm cells are significantly higher in CD4^+^ TILs than in CD8^+^ TILs (28). This pattern aligns with the observed enrichment of Tem cells in tumor-associated lymph nodes, indicating that effector memory differentiation represents a common characteristic of local immune remodeling within both nodal and intratumoral compartments.

Collectively, these findings highlight the compartment-specific distribution of memory T-cell subsets across peripheral blood, tumor-associated lymph nodes, and tumor tissues. However, it remains unclear whether these shifts reflect effective antitumor adaptation or altered cellular trafficking and progressive functional exhaustion.

In addition to quantitative changes, Tcm and Tem cells in lung cancer exhibit functional alterations at the levels of cytokine production and gene expression. In peripheral blood, CD4^+^ Tcm/Tem and CD8^+^ Tem cells show reduced levels of IFN-γ, TNF-α, and IL-17 compared with healthy controls (20). Although CD8^+^ Tcm cells in tumor-involved lymph nodes produce increased IFN-γ, their overall function remains impaired (20). TILs contain a higher proportion of cytokine-producing cells, with up to 72.06% of CD8^+^ Tem cells expressing IFN-γ (28). Single-cell RNA sequencing further reveals the downregulation of quiescence genes (TCF7, LEF1), upregulation of activation markers (CD95, GZMK), and reduced clonal diversity in CD8^+^ Tcm cells (18). At the transcriptomic level, CD4^+^ Tcm cells upregulate angiogenesis-related pathways while downregulating immune signaling pathways, including NF-κB signaling, whereas CD4^+^ Tem cells show reduced expression of cytotoxic genes but increased expression of pro-inflammatory factors, together indicating a shift toward dysfunctional and potentially pro-tumoral states (80). Similar dysfunctions have also been described in other malignancies. In head and neck squamous cell carcinoma, Tcm cells decrease in frequency and show weakened Bcl-2-mediated survival, whereas Tem cells exhibit increased apoptosis (89). In malignant melanoma, CD8^+^ Tem cells are markedly reduced in frequency, yet the residual population displays an activated phenotype (CD38^+^HLA-DR^+^), whereas CD4^+^ Tcm cells acquire an exhausted phenotype characterized by high PD-1 expression (83). Collectively, these findings demonstrate that cancer-associated alterations in memory T cells extend far beyond numerical redistribution, encompassing profound functional reprogramming characterized by compromised effector competence, dysregulated survival homeostasis, and the emergence of dysfunctional or terminally exhausted cellular states.

Overall, current evidence demonstrates substantial quantitative and functional remodeling of Tcm and Tem cells across the peripheral circulation, lymphoid tissues and tumor sites in NSCLC. Although the precise alterations vary across tissue compartments, disease stages and experimental approaches, prevailing evidence indicates a global redistribution rather than uniform expansion or contraction of individual memory T−cell subsets, accompanied by pervasive functional impairment. Such re-modeling compromises endogenous anti-tumor immunity and harbors considerable potential for clinical application as prognostic and immunotherapeutic biomarkers.

## The distribution and function of Trm cells

Tissue-resident memory T cells are important mediators of immune surveillance in peripheral tissues, enabling rapid local responses upon antigen re-exposure and contributing to antitumor immunity. Their development remains incompletely understood, and two primary models have been proposed. The “local divergence” model suggests that circulating memory precursor T cells acquire tissue-residency traits within non-lymphoid tissues (NLTs) through signals such as TGF-β and IL-15 (90–92). In contrast, the “systemic divergence” model posits that events occurring in lymphoid tissues or the circulation direct some precursors toward a Trm cells fate prior to tissue entry (90, 93, 94). These models are not mutually exclusive, as both systemic and local signals are likely to contribute to Trm cells differentiation.

Trm cells persist in NLTs such as mucosal barriers, skin, the gastrointestinal tract, and solid organs, where they persist long term and respond rapidly to antigen re-exposure (95–97). Their residency is regulated by transcription factors such as Runx3 and Hobit, which suppress the egress receptors S1PR1 and enhance the expression of retention markers such as CD69 and CD103 (95, 98, 99). These adaptations enable Trm cells to form a tissue-based surveillance network that supports ongoing immune surveillance and rapid effector responses upon pathogen encounter.

## The impact of aging on Trm cells

Trm cells undergo a tightly coordinated, stage-specific changes in both abundance and function throughout the lifespan (**Figure 2**). During infancy and childhood, Trm cells rapidly accumulate and functionally mature in a site-specific manner within barrier tissues such as the lung and intestine, thereby establishing the foundational tissue-resident memory pool (100, 101). This is followed by a prolonged period of adult homeostasis, approximately 20–65 years of age, during which the Trm cells repertoire maintains clonal diversity and tissue-specific distribution with relatively limited age-related change, consistent with its long-term persistence (88, 89). In older adults, the final phase is characterized by an uncoupling of cellular persistence from protective function, as Trm cells exhibit significant functional impairment, contributing to age-associated immune decline (102–105).

These age-associated changes are reflected across distinct tissue compartments. In the intestinal mucosa, Trm cells numbers remain relatively stable in older adults, but their function becomes impaired, as evidenced by a reduced capacity to produce key cytokines, such as IL-17A and IL-2 in response to challenge, including oral vaccination (103, 104). In the lung, the paradoxical numerical expansion of CD8^+^ Trm-like cells in older individuals coincides with hallmark features of dysfunction, including a constricted TCR repertoire, elevated expression of exhaustion markers such as PD-1, and reduced protective capacity (105). Collectively, these findings underscore that advancing age erodes the functional integrity—rather than the mere persistence, of the Trm pool.

## The composition and function of Trm cells in NSCLC

Trm cells constitute a critical subset of tumor-infiltrating lymphocytes that persist in peripheral tissues and mediate local immunity across multiple cancer types (106). In NSCLC, CD8^+^ Trm cells, characterized by the canonical CD103^+^CD69^+^CD49a^+^ phenotype, represent a pivotal and abundant immune population, accounting for up to 51% of CD8^+^ tumor-infiltrating lymphocytes in early-stage disease (107). These cells are strategically concentrated within key immunological niches and show pronounced accumulation at the invasive margin, where their density strongly correlates with improved patient survival (107–109). Moreover, a high baseline density of CD103^+^ Trm cells serves as a strong, independent predictor of response to immune checkpoint blockade (ICB) (107, 110). Additionally, Trm cells populations are dynamically modulated by therapy; for instance, neoadjuvant chemotherapy has been reported to significantly expand both CD8^+^ and CD4^+^CD103^+^ Trm subsets (109).

NSCLC Trm cells constitute a functionally heterogeneous population rather than a uniformly protective subset. While PD-1^+^TIM-3^-^ Trm cells retain cytotoxic potential and are associated with favorable clinical outcomes, PD-1^+^TIM-3^+^ Trm cells display features of dysfunction and correlate with poorer prognosis (108, 111). These findings indicate that inhibitory receptor expression reflects distinct functional states rather than a uniform exhausted phenotype. In parallel, CD103^+^CD49a^+^ Trm cells appear to define a particularly potent effector subset and have been reported to predict response to anti-PD-1 therapy more accurately than other biomarkers (112). Notably, even though Trm cells express multiple inhibitory receptors, including PD-1, CTLA-4, TIGIT, and CD39, they can retain cytotoxic activity through granzyme production and CD103–E-cadherin interactions (107, 110, 113, 114), suggesting that exhaustion-associated phenotypes do not necessarily indicate terminal functional impairment. Their antitumor activity is further shaped by the tumor microenvironment: chemokine axes such as CXCR6/CXCL16 and CXCL13/CXCR5 regulate intratumoral localization, whereas abnormal angiogenesis may limit infiltration, providing a rationale for combining immunotherapy with anti-angiogenic strategies (111, 113, 115). Collectively, these findings suggest that the contribution of NSCLC Trm cells to antitumor immunity and immunotherapy response depends not simply on their presence, but on their functional state, spatial organization, and microenvironmental context. Across both physiological aging and the tumor setting, therefore, the protective value of Trm cells is defined less by their persistence in tissues than by the preservation of their functional competence and appropriate spatial positioning.

## Discussion

The preceding sections have delineated the distribution, heterogeneity, and regulatory mechanisms governing long-lasting T cell subsets under both physiological conditions and in NSCLC. These phenotypic and functional patterns further uncover profound alterations in T cell biology shaped by aging and tumor-derived signals, which have significant implications for antitumor immunity. Collectively, immunosenescence in lung cancer emerges as a multifaceted process driven by intrinsic T cell dysfunction, age-associated remodeling of the tumor microenvironment, and dysregulated cellular metabolism, all of which converge to impair antitumor immune responses.

At the cellular level, this process is partially attributable to intrinsic defects within the T-cell compartment. Aging diminishes thymic output, restricts peripheral T cell replenishment, and narrows the T cell receptor (TCR) repertoire, thereby limiting the pool of potentially tumor-reactive clones (116). Beyond this quantitative decline, aging also disrupts the stem-like and memory-associated transcriptional programs that sustain T cell persistence, self-renewal, and developmental plasticity, especially in Tscm and progenitor-like memory subsets (46, 117, 118). Consequently, the aged T cell compartment progressively skews toward senescence and terminal differentiation, exhibiting reduced proliferative capacity and impaired effector plasticity. At the molecular level, this phenotypic shift is partially driven by the age-associated downregulation of TCF1 and the epigenetic repression of its encoding gene, TCF7, thereby impairing the maintenance of progenitor-like, long-lived, and therapy-responsive T cell states (118). About the epigenetic and transcriptional mechanisms regulating the frequency and function of Tscm cells need to be further clarified, and whether targeting Tscm cells can reverse age-related immunosenescence requires more preclinical and clinical evidence.

However, these cell-intrinsic defects do not occur in isolation; rather, they are reinforced by broader age-associated perturbations in the immune and stromal microenvironments. Aging is accompanied by a systemic pro-inflammatory state characterized by chronic low-grade inflammation and the accumulation of senescent stromal cells that secret senescence-associated secretory phenotype (SASP) (119). Concurrently, aging facilitates the expansion of dysfunctional or immunosuppressive T-cell populations, such as CD39^+^CD73^+^CD8^+^ T cells, which develop via a B cell–dependent pathway and strongly inhibit antitumor immunity within the tumor microenvironment (120). Beyond these systemic age-related alterations, the aged tumor microenvironment further impairs immune priming and local immune surveillance while favoring the accumulation of tumor-infiltrating, age-associated dysfunctional T cells, collectively creating a permissive niche for cancer progression (121). It remains unreported whether excessive immunosuppressive cytokines (e.g., IL-6, TGF-β) and reactive oxygen species (ROS) secreted by senescent immune cells—including senescent T cells, macrophages and dendritic cells—can inhibit the activation and proliferation of functional Tscm and memory T cells in NSCLC. This potential mechanism may provide novel insights into the underlying immunoregulatory pathways.

Metabolism plays a central role in T-cell immunity, with nutrients serving as a “fourth signal” that works alongside antigen, co-stimulatory, and cytokine signals to regulate T-cell activation, differentiation, and function (122). Metabolic dysregulation in the tumor microenvironment induces T-cell dysfunction, thereby impairing anti-tumor immune function. Study has shown that aging impairs antitumor immunity by disrupting glycolytic and mitochondrial metabolism in CD8^+^ T cells (119). In addition, age-related metabolites, such as methylmalonic acid (MMA), may further inhibit CD8^+^ T-cell function by impairing the TCA cycle and oxidative phosphorylation, as well as by inducing expression of the transcription factor TOX (123). Collectively, these metabolic disturbances likely reduce the capacity of aged T cells to adapt to the nutrient-deprived tumor microenvironment. Within tumors, malignant cells exacerbate this challenge by consuming key nutrients like glucose and glutamine, thereby restricting metabolic support for T cells and promoting inhibitory receptor expression and exhaustion-associated programs (124). The specific molecular targets within metabolic pathways that can be exploited to restore anti-tumor immunity in elderly NSCLC patients remain limited, requiring further preclinical studies to validate.

Convergent cellular, environmental and metabolic changes collectively shape prominent age-dependent disparities in the tumor immune landscape of lung cancer. Rather than chronological age alone, these differences are better understood through variations in immune contexture. Integrated single-cell and transcriptomic analyses reveal that NSCLC in older individuals exhibit a highly infiltrated yet immunosuppressive microenvironment, characterized by increased Treg-cell infiltration, diminished functional NK cells, effector CD8^+^ T cells, and γδ T-cell states, along with elevated immunosenescence scores (125, 126). Consistent with these intratumoral alterations, analyses of peripheral blood from elderly patients have uncovered aging-associated immune imbalances, including reduced proportions of CD3^+^ T cells and increased frequencies of CD14^+^ monocytes (127). In contrast, early-onset lung cancer is characterized by a less immune-infiltrated phenotype, marked by downregulation of immune- and metabolism-related gene signatures and reduced intratumoral infiltration of CD3^+^, CD4^+^, and CD8^+^ T cells—a feature that may underlie the poorer responses to immune checkpoint inhibitor (ICI) therapy observed in this patient cohort (128). Collectively, these findings indicate that elderly patients more frequently exhibit an immunosenescent and suppressive tumor microenvironment, whereas younger patients tend to display reduced T-cell infiltration and diminished pre-existing antitumor immune activation, both of which may influence differential responses to immunotherapy.

Aging is associated with T-cell dysfunction and broader alterations in systemic immunity and the tumor microenvironment; however, these changes do not uniformly translate to poorer immunotherapy outcomes in older patients. Emerging evidence indicates that age-related immunological alterations are not consistently linked to reduced benefit from immune checkpoint inhibitors (ICIs) and, therefore, should not independently preclude older individuals from receiving these therapies (129, 130). In most studies, patients aged 65 years and older demonstrated overall survival benefits comparable to those observed in younger cohorts, indicating that chronological age alone is not an independent predictor of immunotherapy efficacy. Nonetheless, some studies have reported a trend toward attenuated benefit in patients aged 75 years or older, particularly with combination regimens such as immunochemotherapy or dual immune checkpoint blockade (131, 132). Although severe immune-related adverse events are not consistently more common in older patients, treatment is more challenging due to impaired functional status, frailty, comorbidities, and complex toxicity management. Therefore, the efficacy of immunotherapy should be evaluated from multiple dimensions to formulate individualized clinical treatment strategies.

Beyond the conventional T-cell compartment, immune remodeling associated with aging and tumorigenesis also impacts innate-like T-cell subsets, thereby adding an additional layer of regulation for antitumor immunity. Emerging evidence indicates that both aging and the lung tumor microenvironment drive remodeling of innate-like T-cell subsets, with profound implications for antitumor immune responses. Peripheral immune profiling shows that γδ T cells and NKT-like cells (CD3^+^CD56^+^) remain relatively stable until approximately 35 years of age, after which their frequencies decline. Notably, the Vδ2 subset decreases to less than 50% of total γδ T cells beyond 45 years of age, reflecting age-related restructuring within this population (133). Aging is further associated with functional alterations in γδ T cells, including reduced clonal diversity, impaired tissue repair capacity, and a shift toward a pro-inflammatory phenotype (134). In NSCLC, the tumor microenvironment induces γδ T-cell exhaustion, a state linked to poor prognosis. Conversely, higher peripheral frequencies of Vδ2^+^ γδ T cells with reduced PD-1 and TIGIT expression, together with increased intratumoral γδ T-cell infiltration, correlate with improved clinical outcomes following chemotherapy or targeted therapy and prolonged recurrence-free survival (135). These observations suggest that age- and tumor-associated remodeling of innate-like T-cell subsets may compromise effective antitumor immunity and contribute to variability in clinical responses.

Collectively, these findings offer a valuable framework for interpreting long-lasting T-cell subsets in lung cancer; however, they also underscore several conceptual and methodological limitations. Although this review is primarily based on human clinical data, much of the mechanistic insight into lung cancer immunity continues to be derived from murine models. While these models have been pivotal in elucidating immune mechanisms, interspecies differences constrain the direct extrapolation of findings from mouse systems to human disease, thereby emphasizing the necessity for complementary approaches such as humanized models, organoids, and patient-derived analyses. Furthermore, the markers discussed herein are not specific to lung cancer but predominantly represent broader T-cell states common across various tumor types. Given that most solid tumors lack universal tumor-specific T-cell markers, tumor-reactive populations are most accurately characterized through an integrated analysis of phenotypic, functional, transcriptional, and spatial attributes.

Metabolic disorders induce T cell dysfunction and promote tumor initiation. Aging-associated immunometabolic dysfunction appears to be a critical mechanism linking T-cell decline to tumor progression and therapeutic resistance. Consequently, targeting metabolic dysregulation may offer a promising approach to delay immune aging, reduce lung cancer risk, and improve immunotherapy outcomes when combined with existing treatments. Further research is warranted to elucidate how aging alters T-cell function and distribution in lung cancer and to identify actionable immunometabolic targets for the personalized management of elderly patients.

## Conclusion

This review summarizes the spatiotemporal distribution, functional heterogeneity, and regulatory features of long-lasting T-cells subsets, including naive, stem cell-like memory, central memory, effector memory, and tissue-resident memory T cells, in physiological conditions and in NSCLC. Taken together, the available evidence indicates that these populations are dynamic and are continually reshaped by ageing, tissue context, and tumor progression, with important consequences for immune homeostasis and antitumor surveillance.

Beyond descriptive changes in subset composition, the evidence suggests that T-cell ageing in lung cancer is a multilevel process involving intrinsic T-cell dysfunction, age-related remodeling of the tumor microenvironment, and immunometabolic disturbance. These interconnected changes may restrict tumor-reactive clonal capacity, impair long-lived and progenitor-like T-cell states, foster dysfunctional immune microenvironments, and contribute to age-related differences in treatment responsiveness. In this setting, chronological age alone is insufficient to explain immune competence or predict immunotherapy benefit; instead, age-related immune contexture, functional status, and metabolic fitness are likely to be more informative.

In-depth exploration of the complex intertwined mechanisms between immune-senescence and lung cancer pathogenesis may thus provide critical insights to inform both preventive and therapeutic strategies. Elucidating how ageing remodels T cell maintenance, tissue tropism, and metabolic adaptation has the potential to refine risk stratification, underpin approaches to preserve antitumor immunity, and guide the development of more precise therapeutic interventions. In particular, integrating human clinical data with mechanistic studies—while focusing on actionable immunometabolic pathways—could facilitate the refinement of patient stratification and enhance the efficacy of immunotherapies, particularly in the elderly population.

## Literature search strategy

Relevant studies were identified through structured searches of PubMed and Web of Science, covering publications from 1992 to February 2026. Search terms were used alone or in combination and included “naive T cells, ” “memory T cells, ” “T stem cell memory, ” “Tscm, ” “central memory T cells, ” “Tcm, ” “effector memory T cells, ” “Tem, ” “tissue-resident memory T cells, ” “Trm, ” “T-cells migration, ” “T-cell trafficking, ” “aging, ” “immune senescence, ” “immunemetabolism, ” “lung cancer, ” “non-small cell lung cancer, ” and “tumor microenvironment.” Priority was given to peer-reviewed English-language studies directly relevant to the development, maintenance, migration, tissue distribution, and functional characteristics of long-lasting T-cell subsets, particularly in the contexts of aging and lung cancer. Foundational studies were included to establish conceptual background, while recent publications were selected to reflect current mechanistic and clinical advances. Additional relevant articles were identified through manual screening of the reference lists of key publications. The final selection was guided by the aim of providing a focused and integrative synthesis of the field.
